# Growth regulation by apyrases: Insights from altering their expression level in different organisms

**DOI:** 10.1093/plphys/kiad590

**Published:** 2023-11-08

**Authors:** Greg Clark, Manas K Tripathy, Stanley J Roux

**Affiliations:** Department of Molecular Biosciences, The University of Texas at Austin, Austin, 100 E 24th Street, TX 78712, USA; Institute of Life Sciences, Bhubaneswar, 751023 Odisha, India; Department of Molecular Biosciences, The University of Texas at Austin, Austin, 100 E 24th Street, TX 78712, USA

## Abstract

Apyrase (APY) enzymes are nucleoside triphosphate (NTP) diphosphohydrolases that can remove the terminal phosphate from NTPs and nucleoside diphosphates but not from nucleoside monophosphates. They have conserved structures and functions in yeast, plants, and animals. Among the most studied APYs in plants are those in Arabidopsis (*Arabidopsis thaliana*; AtAPYs) and pea (*Pisum sativum*; PsAPYs), both of which have been shown to play major roles in regulating plant growth and development. Valuable insights on their functional roles have been gained by transgenically altering their transcript abundance, either by constitutively expressing or suppressing *APY* genes. This review focuses on recent studies that have provided insights on the mechanisms by which APY activity promotes growth in different organisms. Most of these studies have used transgenic lines that constitutively expressed *APY* in multiple different plants and in yeast. As APY enzymatic activity can also be changed post-translationally by chemical blockage, this review also briefly covers studies that used inhibitors to suppress APY activity in plants and fungi. It concludes by summarizing some of the main unanswered questions about how APYs regulate plant growth and proposes approaches to answering them.

## Teaser

The genetically enhanced expression of certain apyrases in yeast and in multiple different plants promotes the growth of these organisms.

## Introduction: early studies link eATP, ecto-apyrase activity, and growth regulation

Plant apyrases [nucleoside triphosphate (NTP) diphosphohydrolases (NTPDases)] were characterized in potato (*Solanum tuberosum*) (Krishnan 1948). They differed from other phosphatases in that their substrates were mainly NTPs, and, although they could hydrolyze nucleoside diphosphates (NDPs), they could not remove phosphates from NMPs. As noted in Clark and Roux (2018), their low *K*_m_ for NTP and NDP nucleotides gave them great value as tools for removing unwanted ATP from biochemical assays (e.g. Ho and Theg 2016). Their importance for plant growth and development did not become evident until the publication of early studies of a pea (*Pisum sativum*) apyrase, which was found to function in diverse subcellular locales to control a variety of cellular processes (Matsumoto et al. 1984; Chen and Roux 1986; Shibata et al. 2002). The pea apyrase had a calmodulin-binding domain, so these cellular processes included signaling changes induced by increased [Ca^2+^]_cyt_, because the binding of Ca^2+^-activated calmodulin to the pea apyrase stimulated its enzyme activity, thus enabling it to transduce Ca^2+^ signals into cellular changes (Chen et al. 1987; Basu et al. 2021). Additionally, because calmodulin has a documented role in nuclear trafficking of proteins including transcription factors (Sweitzer and Hanover 1996; Hanover et al. 2009), it could play a role in targeting apyrase to the nucleus. The initial pea apyrase studies focused only on the potential of the enzyme to regulate the concentration of ATP inside the cell and induce changes there.

Subsequent discoveries broadened the scope of apyrase functions to include limiting the concentration of extracellular ATP (eATP). Although an early report suggested the possibility that eATP could function as a signaling agent in plants (Udvardy and Farkas 1973), it has been only within the last 2 decades that a continuous stream of publications every year has provided definitive evidence for this conclusion. These reports, including critical data on plant eATP receptors (Choi et al. 2014; Pham et al. 2020), raised the question of how plants control the level of extracellular nucleotides. The answer to this question was already evident in animal cells more than 25 years ago, when some apyrases were classified as ecto-apyrases (ecto-NTPDases); i.e. apyrases that function in the extracellular matrix (ECM) to remove NTPs and NDPs from this domain. Because ecto-apyrases had the lowest *K*_m_ among the known ecto-phosphatases that could hydrolyze eATP, they were accepted as being critically important in limiting the concentration of NTPs and NDPs in the ECM that could activate the purinergic receptors known to control diverse responses in animal cells (Zimmermann 1994). Apyrase enzymes are highly conserved throughout evolution (Clark et al. 2014), so it was not surprising when multiple reports indicated that ecto-apyrases also played a major nucleotide-hydrolyzing role in the ECMs of diverse plants, such as Arabidopsis (*Arabidopsis thaliana*) (Wu et al. 2007), soybeans (*Glycine max*) (Govindarajulu et al. 2009), potato (Riewe et al. 2008), and poplar (*Populus euphratica*) (Deng et al. 2015).

Initially, the most studied apyrases linked to growth control were the pea enzyme, psNTP9 (hereafter abbreviated PS) and 2 almost identical apyrases in Arabidopsis, AtAPY1 and AtAPY2 (abbreviated as AtAPY1/2 when both may be involved). Early evidence indicated that at least some portion of both the pea and Arabidopsis enzymes could be localized in the ECM and could thus qualify as ecto-apyrases (Thomas et al. 1999; Shibata et al. 2002; Wu et al. 2007). Another feature of both apyrases that could involve them in growth control is that they bind to calmodulin (Chen et al. 1987; Steinebrunner et al. 2000).

When purified to near homogeneity from etiolated seedlings, both PS and AtAPY1/2 favor ATP as their substrate, and have the same vanadate insensitivity characteristic of most animal and plant apyrases (Chen et al. 1987; Steinebrunner et al. 2000). However, the substrate specificity of apyrases can be readily altered by biochemical modifications (Knowles 2011). As noted above, untagged PS and AtAPY1/2 directly purified from dark-grown seedlings favor ATP as their NTP substrate; however, tagged AtAPY1 purified from light-grown cells favors ADP as its substrate (Chiu et al. 2015; Massalski et al. 2015). The molecular bases of these differences in substrate specificity remain to be discovered, but it seems likely that, at least in Arabidopsis, endogenous AtAPY1 can function as an ecto-apyrase to limit [eATP], as demonstrated for pollen AtAPY1/2 (Wu et al. 2007), and in tissue culture of seedlings suppressed in their expression of APY1/2 (Lim et al. 2014). In both these cases, inhibiting apyrase expression or activity led to an increase in [eATP].

Early studies showed that the “ecto” role of apyrases included growth regulation (Thomas et al. 1999; Steinebrunner et al. 2003). How cell wall/ECM NTPDase activity could be linked to growth became clear when dose–response assays showed that low levels of eATP could promote growth and high levels could suppress it (Clark et al. 2010a, 2010b). Correspondingly, the apyrase-growth connection was found to have a hormonal basis when Tang et al. (2003) found that high [eATP] could inhibit auxin transport, Shi et al. (2022) documented that jasmonic acid signaling could mediate the inhibitory effects of raised eATP levels on the growth of Arabidopsis seedlings, and transgenic studies showed that enhanced expression of *AtAPY1* or *AtAPY2* could promote auxin transport while their suppression inhibited this transport (Liu et al. 2012).

### Main goals of this review

Given the above background information, this review will serve as an update on the latest findings on plant apyrases. It will supplement and extend 2 other recent reviews, one on the role of apyrases in regulating eATP signaling (Clark and Roux 2018), and the other on apyrase structure and function (Clark et al. 2021), both of which mainly cited literature published before 2021. Thus, the main goals of this review will be to update readers on the progress made on these same topics during the last 2+ years, and to introduce other results that are especially relevant to an understanding of the mechanisms by which apyrases regulate growth in different organisms. Of course, for the sake of completeness and context, we will also comment on how the recent data relate to those of prior studies.

### Apyrase and plant growth responses to wounding and biotic attacks

The link between eATP, apyrase activity, and growth extends also to plant responses to wounding and insect or pathogen attacks, in which a key early event after is the release of cellular ATP into the ECM through broken or permeabilized membranes. This increase in eATP serves as a signal to turn on defense genes (Tanaka and Heil 2021), and jasmonic acid signaling pathways (Shi et al. 2022) that inhibit growth, but it also induces an increase in apyrase expression, as discussed in Clark et al. (2021).

Recent RNA-seq data reported in the Sequence Read Archive data base of the NIH National Library of Medicine (accession number SRX3943120) indicate that defense-related genes are the ones whose expression is most upregulated in transgenic plants that constitutively expressed AtAPY1. These included the *TGG2* gene, one of only 3 genes upregulated more than 80-fold. It encodes one of the thioglucoside glucohydrolases that is released from herbivore-inflicted wounds. As reported recently by Gao et al. (2023), these enzymes function as factors (called “Ricca” factors) that provoke long-distance electrical waves that move to distant undamaged sites, where these signals induce defense responses needed to protect against anticipated future attacks from herbivores (Gao et al. 2023). Hedrich and Roelfsema (2023) review how the activity of TGGs leads to the cleavage of glucosinolates, the release of glutamates into the xylem, and the activation of glutamate-like receptors known to be key for the systemic transmission of wound-induced electrical waves. However, additional studies would be needed to clarify if and how the TGG signal can protect the entire plant from herbivore attack.

Although the constitutive expression of AtAPY1 in Arabidopsis strongly upregulates defense-related genes, and a poplar apyrase is upregulated by wounding (Major and Constabel 2006), there are currently no reports that directly support the hypothesis that upregulating apyrase expression is a key event induced by eATP to elicit the defense responses of plants to pathogen or herbivore attack. This hypothesis could be tested by examining the effects of apyrase suppression on the effectiveness of eATP as a damage-associated molecular pattern (DAMP) signal.

### Plants have multiple apyrases, and different apyrases impact plant growth differently

A favored approach that plant scientists use to evaluate the function of a protein in any specific plant is to either constitutively express it or genetically knock it out in that plant. However, discovering which of the apyrases in a plant are specifically ecto-apyrases is challenging, because most plants have multiple apyrases, most of which function inside the cell. For example, soybean has 13 different apyrases, but so far only GS52 has been identified as an ecto-apyrase (Govindarajulu et al. 2009), and potato has 10 different apyrases, but so far only 3 of these have been shown to function in the ECM (Riewe et al. 2008). Similarly, not all apyrases in a plant stimulate growth when constitutively expressed, or suppress growth when genetically suppressed. For example, of the 7 apyrases in Arabidopsis, initial transgenic studies favored only AtAPY1 and AtAPY2 as having critically needed roles in growth control (Wolf et al. 2007; Wu et al. 2007; Yang 2011). So far, the apyrases that have been reported to regulate growth in Arabidopsis, soybeans, potato, peas, and poplar all have the plasma membrane/ECM as one of their major locales, so all could potentially function as ecto-apyrases (Clark et al. 2021). Thus, the ecto-apyrase-eATP-growth regulation link seems to be a consistent theme in plants.

To the extent that only some of the apyrases in a plant play major roles in growth control, it would be valuable to identify these specific apyrases in other plants, especially in crop plants. A relatively rapid RT-PCR assay that could be used for this purpose was illustrated in Wu et al. (2007). In that report, the transcript levels of *AtAPY1* and *AtAPY2* were shown to be strongly correlated with growth rates of tissues in seedlings. That is, they were highly expressed in hypocotyls that are rapidly growing in darkness, but almost absent from the same tissue when its growth is rapidly suppressed by exposure to light. Thus, in the scores of plants for which the genomic sequences are now known, it would be straightforward to design primers to quickly assay by RT-PCR which apyrase transcripts in the hypocotyl or epicotyl of a given dicot seedling, or in the rapidly growing mesocotyl of a monocot like maize dramatically change expression when the growth of these tissues rapidly declines as they transit from darkness into light. Of course, the success of this strategy would depend on whether the specific growth-regulatory apyrases in the assayed plant are preferentially expressed in rapidly growing tissues, and it remains to be seen how many other plants follow the pattern seen in Arabidopsis.

## Recent studies linking increased expression of native apyrase in Arabidopsis to enhanced growth

Beyond the evidence reviewed by Clark et al. (2021), more recent publications have provided insights on the apyrase-growth control connection. As summarized and discussed further here, these reports revealed ways in which enhancing or diminishing the expression of this enzyme can induce changes in plants’ gene expression and in their growth and development.

### Endophytic bacteria induce AtAPY5 production in Arabidopsis and increase growth

Plants and microorganisms interact in multiple ways, some of which are beneficial for both.

Multiple reports have demonstrated that some microorganisms that live in plants can promote their growth and seed yields, and thus can be used as biofertilizers. Endophytic *Bacillus* species that can survive in adverse environments are among the microorganisms most used in this way, and some have been shown to produce metabolites that promote plant growth and increase crop production (Ke et al. 2021).

A recent report demonstrated that endophytic bacteria can influence growth by a mechanism distinct from just producing nutrients for plants (Xu et al. 2022). This report showed that the interaction of the endophytic bacterium *Bacillus aryabhattai* with Arabidopsis and tobacco (*Nicotiana tabacum*) plants resulted in a statistically significant, 2-fold increase in their growth, as measured in both dry and fresh weight. In Arabidopsis, its co-incubation with the endophyte induced it to turn on the transcription of multiple genes that stimulate growth (Xu et al. 2022). The second highest fold-increase in transcript abundance (over 2,000-fold) was for the transcript encoding AtAPY5. Based on this fact, the authors hypothesized that this apyrase functioned in some key role that influenced growth. However, this role was not very obvious because a previous study had shown that null *atapy5* mutants had no clear phenotype that differed from wild-type plants (Yang 2011). Interestingly, *AtAPY5* expression was increased almost 5-fold in *APY1*-overexpressing (*APY1*-OE) seedlings, which showed improved growth when grown on Pi-sufficient, NTP supplemented media (Slocum et al. 2023). AtAPY5 is predicted to be Golgi localized and have a high activity toward NDPs (Meng et al. 2019). Although AtAPY5 may not play an essential role in growth regulation, its enhanced expression could still promote growth.

One hint of how AtAPY5 could have promoted growth of Arabidopsis and tobacco is that it can complement a yeast mutant that is defective in cell wall synthesis (Chiu et al. 2015). Several of the other genes whose transcript abundance was upregulated by the endophyte were known to promote lignin synthesis, a change that would certainly impact plant growth (Liu et al. 2018), so further studies on the role of AtAPY5 in the production of this cell wall component would be warranted. Further, altering expression of Golgi-localized apyrases could affect growth by altering glycosylation levels of proteins which play a role in cell growth (Veit et al. 2015, 2018).

### APYRASE1/2 play a critical role in mediating light-induced growth changes in Arabidopsis

As noted above, when etiolated seedlings emerge from darkness into light, the activation of phytochrome induces major changes in gene expression and in growth. The growth changes differ in different tissues: hypocotyl growth rate drops dramatically and quickly, while that of roots and cotyledon-hook tissue increases somewhat more slowly (Montgomery 2016). The results of Wu et al. (2007) demonstrated that the strong and rapid decrease in the growth of the hypocotyl occurred with the same rapid kinetics as an equally dramatic decrease in both the transcript and protein levels of AtAPY1 and AtAPY2. This raised the question whether the transcript and protein levels of AtAPY1/2 in the root and hook-cotyledon tissue of seedlings also change when light induces their increased growth during de-etiolation, and, if so, whether these changes were needed for the growth changes. The answers to these questions turned out to be Yes, as reported in Weeraratne et al. (2022), and as described and discussed here.

The growth changes in primary roots induced by red light (R) are not unidirectional, for roots initially grow slower during the first 12 h after irradiation (Correll and Kiss 2005), but then, at later time points, they grow faster (Kircher and Schopfer 2012). At the same time points, after 12 h of light AtAPY protein levels decreased, but after 24 h the levels of both *AtAPY* transcripts and protein increased.

Unlike the growth response of roots to light, hook-cotyledon tissue shows a steady, consistent increase in growth, and immunoblot assays showed that the kinetics of AtAPY protein increase in these tissues coincided with their growth increase. However, in this case, *AtAPY* transcript levels did not increase in parallel, indicating that in these aerial tissues R increased AtAPY expression mainly by increasing its translation and/or decreasing its turnover (Weeraratne et al. 2022). Other instances of phytochrome inducing changes in gene expression mainly by controlling translation are known (Paik et al. 2012). Specifically, in etiolated seedlings, Cheng et al. (2021) recently reported that R-induced changes in the level of certain proteins occurred without parallel changes in the transcripts that encode those proteins.

A well-studied morphological change that occurs during R-induced de-etiolation is hook opening. Weeraratne et al. (2022) found that RNAi-mediated suppression of *AtAPY1/2* expression severely inhibited hook opening, while the constitutive expression of either *AtAPY1* or *AtAPY2* could induce this opening even in unirradiated etiolated plants. These results were consistent with the hypothesis that changes in *AtAPY1/2* expression could help mediate the transcriptomic changes known to be required for this tissue-straightening response to occur. These included the upregulation of a Small Auxin Up-Regulated (SAUR) gene, *SAUR50* (Dong et al. 2019). This, in turn, required the downregulation of *SAUR17* (Wang et al. 2020). In support of this hypothesis, Weeraratne et al. (2022) found that the over-expression of *AtAPY2* promoted both the upregulation of *SAUR50* and the downregulation of *SAUR17* in the hook-cotyledon tissue of etiolated seedlings. Taken together, these results implied that in the sequence of transcriptomic changes that occur when phytochrome induces hook opening, the upregulation of the genes *AtAPY1* and *AtAPY2*, both of which have promoter elements known to be regulated by phytochrome (Wu et al. 2007), precede the R-induced changes in the *SAUR50* and *SAUR17* genes.

Other transcriptomic changes that impact growth, and that occur in dark-grown seedlings of transgenic Arabidopsis plants in response to enhanced or suppressed expression of AtAPY1/2 included changes in the transcript abundance of 3 ECM peroxidases, Prx15, Prx49, and Prx59 (Table 1). The activity of these peroxidases catalyzes crosslinks in cell walls that decrease their extensibility (Lim et al. 2014). The constitutive expression of *APY2* decreased the transcript level of all 3 of these peroxidases while the RNAi-mediated suppression of AtAPY1/2 increased the level of the same peroxidases (Weeraratne et al. 2022). These results further confirmed the role of AtAPY1/2 in regulating the expression of genes that control growth.

**Table 1.** Examples of the change in expression of growth-regulating genes induced in Arabidopsis by constitutive expression of AtAPY1, or AtAPY2

Growth-regulating gene	*APY*	ΔExpression	Induced growth change	Ref.
*MRN1* (Marneral synthase)	*AtAPY1*	+ 4-fold^b^	Increased meristematic growth	**a**
β–glucosidase 37	*AtAPY1*	+ 83-fold^b^	DAMP-mediated growth	**b**
*ABCB15*	*AtAPY1*	+ 2.8-fold^b^	IAA transport; lateral root growth	**c**
*SAUR50*	*AtAPY2*	+ 2-fold^c^	Cotyledon expansion	**d**
3 wall peroxidase genes^a^	*AtAPY2*	− 2-fold^c^	Increased wall extensibility	**e**

+, increased expression; −, decreased expression.

References: **a,** Go et al. (2012); **b**, Gao et al. (2023); **c**, Chen et al. (2023); **d,** Dong et al. (2019); **e,** Lim et al. (2014); **e**, Kubeš et al. (2012).

^a^ *Prx15, Prx49, Prx59*.

^b^Transcriptomic data reported in SRX3943120 of sequence read archive data base of NIH.

^c^Or transcriptomic data reported in Weeraratne et al. (2022).

### AtAPY1 expression can help salvage phosphate from extracellular NTPs

In another recent report, which is now under review, but is available as a preprint online, Slocum et al. (2023) investigated whether another function of apyrase could be the salvaging of phosphate (Pi) from extracellular nucleotides, such as those known to be present in the rhizosphere from diverse sources. In their study of Arabidopsis seedlings that constitutively express *APY1* (*APY1*-OE), they found that under growth conditions in which Pi availability was limiting, both wild-type and *APY1*-OE seedlings showed the typical Pi starvation response of having decreased Pi contents and a characteristically altered root system architecture (RSA). However, when grown on Pi-sufficient media, *APY1*-OE seedlings had higher Pi contents than wild-type seedlings. Moreover, the addition of NTP increased the Pi contents and expanded the RSA of *APY1*-OE but not that of wild-type seedlings.

Consistent with their elevated Pi contents, *APY1*-OE seedlings showed greater repression of phosphate-starvation-response genes relative to wild-type seedlings, and their expanded RSA was correlated with the increased expression of hundreds of growth-related genes, such as marneral synthase (Go et al. 2012; Table 1), including over 100 involved in regulation of auxin signaling and transport, such as ABCB15 (Chen et al. 2023; Table 1). The authors concluded that APY1 could modulate this auxin response by promoting increased Pi uptake, including some Pi salvaged from extracellular NTPs (Slocum et al. 2023). The data in this report could have potential value for the development of transgenic crops that have higher fertilizer use efficiency and, consequently, higher seed yields. Also, by salvaging more of the Pi applied in fertilizers, those crops overexpressing *AtAPY1* could reduce the environmental pollution due to phosphate run-off from soils.

### Growth effects of APY1/2 may be mediated by their ECM, Golgi, and/or nuclear activities

The “ecto” location of apyrases (i.e. either in the ECM or on the plasma membrane with its active site facing to the ECM) in several different plants has been demonstrated (Clark et al. 2021). Although multiple studies have shown that by modulating eATP levels, ecto-apyrases could alter the growth rate of plant cells, AtAPY1 and AtAPY2 have been localized not only in the ECM, but also in Golgi (Chiu et al. 2012; Schiller et al. 2012) and in nuclei (Weeraratne 2019), and, as discussed by Veerappa et al. (2019), they could impact growth by their activities in any of these organelles. Massalski et al. (2015) previously discussed how the NDPase activity of AtAPY1 in Golgi could impact protein glycosylation, a step typically needed to insert key growth-regulating enzymes into membranes (Veit et al. 2018; Chen et al. 2020).

While there is good evidence for the ECM and Golgi locales of different plant apyrases, the best studied apyrase in peas, PS, was originally purified from pea nuclei (Chen et al. 1987), and it was localized in this organelle by immunocytochemistry (Tong et al. 1993). The 2 most highly expressed apyrases in rapidly growing cells of Arabidopsis, AtAPY1 and AtAPY2 (Wu et al. 2007), also co-localize with purified nuclei and have been immunolocalized there in situ (Weeraratne 2019). Moreover, PS binds to DNA-affinity columns with high specificity (Chen 1987). These findings raise the obvious possibility that AtAPY1/2 and PS could have major functions in the nucleus as well as in the ECM.

Beyond the most studied roles of ATP in various nuclear functions (e.g. transcription, epigenetic modifications, pre-RNA splicing), it also plays a key role as a chromatin hydrotrope and in maintaining nuclear phase–phase separations (Wright et al. 2019). Moreover, a recent study found a role for eATP in regulating chromatin dynamics and chromatin binding proteins in Arabidopsis (Matzke et al. 2019). This is an especially relevant finding because both PS and AtAPY1/2 have been demonstrated to be chromatin-associated proteins (Hsieh et al. 2000; Weeraratne 2019).

Given all these major roles of nuclear [ATP], it would not be surprising to learn that any changes in the nuclear levels of *apyrase* in transgenic plants would dramatically alter the regulation of gene expression. Although none of the transgenic studies (either suppression or over-expression) carried out thus far have tried to assay how the altered levels of apyrase are differently distributed in the nucleus, ECM or Golgi, there is strong evidence that some fraction of AtAPY1/2 is in the nucleus, at least in etiolated seedlings (Weeraratne 2019). Thus, one could expect that some of the effects of altered expression of AtAPY1 in Arabidopsis on changes in the expression of genes that impact growth, such as those revealed by Lim et al. (2014) and Weeraratne et al. (2022), would be due to changes in its nuclear activities, independent of how it impacted NTP levels in the ECM or Golgi.

In parallel with studies of the role of apyrase in modulating transcriptomic changes, other reports have shown that eATP also regulates changes in gene expression. The Redox Responsive Transcription Factor, RRTF, was identified as being eATP-responsive and important in Arabidopsis growth and defense responses (Dong et al. 2020; Zhu et al. 2020). Additionally, MYC transcription factors and a calmodulin-binding transcription activator (CAMTA3) play important roles in mediating eATP-induced changes in gene expression (Jewell et al. 2019; Jewell and Tanaka 2019). Thus, apyrase control of ATP levels in the ECM could also impact nuclear activities.

Two different approaches are currently being used to help resolve which phenotypic and/or gene expression changes induced by altered expression of apyrase are due to which subcellular domain of apyrase function. In one, the nuclear functions of apyrase are being clarified both by ChIP assays, to determine with what (if any) regions of DNA apyrase interacts, and by co-IP and yeast-two hybrid assays to determine with which (if any) chromatin/nuclear proteins apyrase associates. In another assay, transgenic Arabidopsis plants are being generated that express altered versions of PS, missing either their nuclear localization signal or their signal peptide. These plants can then be studied to determine how they differ from transgenic plants expressing wild-type PS in their phenotype and in which genes they differentially express.

## Recent reports on the ectopic expression of apyrases in different organisms further clarify their biochemical and molecular functions

The signal peptide and nuclear localization signals of apyrases could, in principle, allow them to enter into ECM or nuclear domains when they are ectopically expressed in organisms other than their native plants. As another valuable approach to gaining a better understanding of the role of apyrases in plant growth and development, multiple laboratories have studied the expression of different apyrases in a heterologous system.

### Expression of plant apyrase genes in bacteria and yeast

Bacteria and yeast are the simplest heterologous cells in which to express a plant apyrase, and this strategy has been used many times in order to characterize the enzyme activity of different apyrases. Characterization of the enzyme activity of a specific plant apyrase, NTP/NDP preferences and inhibitor assays, are important first steps toward understanding their cellular functions (Handa and Guidotti 1996). Post-translational modifications such as glycosylation and phosphorylation as well as disulfide bond mediated oligomerization are well documented to regulate activity of animal apyrases (Knowles 2011) and thus would be expected to also play critical roles in regulating activity of plant apyrases.

Early studies heterologously expressed apyrases in bacteria to study their biochemical properties. Their results showed that the bacterial expression of both AtAPY1 (Steinebrunner et al. 2000) and a potato apyrase (Wujak et al. 2013) resulted in the recombinant proteins accumulating in inclusion bodies as insoluble proteins, most likely due to their toxic effect on cellular ATP levels or improper folding due to their expression in procaryotic cells. Nonetheless, these apyrases retained strong NTPDase activity after being solubilized and refolded. In a follow-up study, 3 potato APYs (StAPY4-StAPY6) were co-expressed with heat shock chaperonins in *Escherichia coli*, and using this approach StAPY5 was produced in a soluble, catalytically active form (Porowińska et al. 2014). More recently, an improved method of purifying apyrase from potato confirmed that it had high specific activity levels for ATP substrates (Karim et al. 2023).

Expression of plant apyrases in yeast instead of in bacteria has the advantage of allowing for eukaryotic post-translational modifications to the heterologously expressed apyrase. Expression of PS in a yeast phosphate-transport mutant NS219 restored the ability of the mutant to take up phosphate (Thomas et al. 1999). The transgenic yeast grew better under both phosphate-limiting as well as phosphate replete conditions. The mechanism by which this heterologous expression of apyrase allowed the yeast mutant to grow better is still not understood. However, its ectopic expression in Arabidopsis increased phosphate content in both seedlings (Thomas et al. 1999) and mature leaves (Veerappa et al. 2017), so further studies on how the enhanced expression of PS in plants promotes the uptake of this critical nutrient is warranted. Multiple other past studies of the ectopic expression of apyrases in yeast (e.g. potato apyrase expression in the methylotrophic yeast *Pichia pastoris* (Nourizad et al. 2003)), and the expression of all 7 Arabidopsis apyrases in a yeast double mutant that lacked endogenous apyrases (Chiu et al. 2015) further illustrated that plant apyrases can function well in foreign cellular environments.

### Ectopic expression of different apyrases in vascular plants

The ectopic expression of PS in Arabidopsis and soybeans resulted in enhancing the growth of both plants. These effects included an improved RSA, increased fresh and dry weights, and higher seed yield under both normal and drought conditions (Veerappa et al. 2019). Multiple independent transgenic Arabidopsis lines were found to have higher root hair density and longer root hairs as well as longer primary roots and more lateral roots under normal growth conditions and during osmotic stress. Detached shoots and leaves from transgenic lines also showed improved water retention during dehydration, and, accordingly, intact plants showed improved growth and survival in response to drought treatment. RNA-seq analyses of Arabidopsis transgenic lines showed differential expression of genes related to these RSA and drought phenotypes.

The expression of PS in a cultivar of the agriculturally important crop, soybean, also resulted in improved traits when these transgenic plants were grown in the greenhouse. These enhancements included improved RSA and increased yield in normal growth and drought conditions. A model was proposed to explain how the phenotypic changes produced by heterologous expression of PS could be impacted by its activity in any of the 3 potential cellular locales (Golgi, ECM, nuclei) in which this apyrase could function (Veerappa et al. 2019).

In a follow-up study, Sabharwal et al. (2022) found that the improved growth and yield phenotypes observed for 3 different transgenic Williams 82 lines when they were grown in the greenhouse were also observed in these same lines when they were grown in multiple field trials. Even elite soybean lines constitutively expressing *PS* exhibited these improved growth and yield features. Specifically, in field-grown transgenic soybeans the typical increase in yield ranged from 10% to 44%. Moreover, the level of increased yield corresponded to the level of PS being expressed.

The *PS*-expressing soybean lines also had leaf traits that would increase their water use efficiency, and would likely contribute to the increased seed yields. These included higher chlorophyll and protein contents, decreased stomatal density, increased cuticle and cell wall thickness in their epidermal cells, and increased trichome density and trichome length.

RNA-seq analyses of transgenic soybean leaves indicated that their altered phenotypes could be explained, in part, by genome-wide gene expression changes induced by the *PS* transgene. A total of 996 genes were differentially expressed. Among them, those associated with fatty acid and wax biosynthesis and cuticle development and nitrogen assimilation genes were upregulated, and those regulating stomatal development were downregulated. Importantly, Western blot analyses confirmed that the PS protein was localized to both the ECM and nucleus in transgenic soybean plants (Sabharwal et al. 2022). As noted above, PS expression in either of these subcellular domains could impact the gene expression changes observed.

In addition to PS, several other plant apyrases have been ectopically expressed in the model plant Arabidopsis in order to gain insights into their function. One such study expressed 2 apyrase genes from the drought-tolerant poplar (*Populus euphratica*) tree species, *PeAPY1* and *PeAPY2*, in Arabidopsis (Zhang et al. 2021). This study found that expression of either of these Poplar apyrase genes resulted in a drought-tolerant phenotype in Arabidopsis seedlings. Just as was observed in *PS*-expressing Arabidopsis (Veerappa et al. 2019), the expression of *PeAPY1* and *PeAPY2* in Arabidopsis resulted in an increased sensitivity to ABA during stomatal closing. The transgenic *PeAPY1*-OE and *PeAPY2*-OE lines also showed enhanced ABA inhibition of light-induced stomatal opening. Additionally, ABA treatment of wild-type seedlings induced upregulation of NADPH-oxidase gene expression (*AtRBOHD* and *AtRBOHF*), confirming the important role that reactive oxygen species (ROS) plays in ABA regulation of stomatal aperture. Notably, this upregulation was greatly increased in the *PeAPY1*-OE and *PeAPY2*-OE transgenic lines compared to in the wild type.

The results of Zhang et al. (2021) were consistent with multiple other reports showing that an increase in ROS levels is an important early signaling step in eATP regulation of growth in wild-type plants (Myers et al. 2022), and that changes in the expression level of apyrases regulates growth. They also further confirmed a key role for PeAPY2 in stress responses that was originally reported by Deng et al. (2015), who showed that *PeAPY2* expression in Arabidopsis allowed for better primary root growth and increased survival to cold-stress treatment.

### Other apyrases involved in growth control

In prior studies on the effects of increasing apyrase expression in Arabidopsis, only AtAPY1 and AtAPY2 promoted growth. However, they are not the only 2 Arabidopsis apyrases involved in growth control. For example, AtAPY6 and AtAPY7 play critical roles in exine development in pollen grains (Yang et al. 2013). More recently, a study by Gupta et al. (2023), initially published as a nonpeer reviewed preprint, suggests that AtAPY7 participates in regulating cell wall composition and can function as a negative regulator of cell growth. AtAPY7 does not hydrolyze eATP and its effects on growth are independent of signaling processes induced by eATP receptors, so it appears to function as an endo-apyrase. Its role in growth control was discovered by a genetic screen that revealed mutations in *AtAPY7* suppressed root hair growth defects found in mutants of the extracellular Leucine Rich Repeat Extensin proteins and FERONIA. It will be important not only to confirm and follow up on these preliminary results, but also to clarify the role of other endo-apyrases in growth control.

## Apyrase interactions with key signaling proteins

As noted above, the expression of PeAPY2 and other plant apyrases substantially impacts eATP and ABA responses, stomatal functions, and vesicular trafficking, all of which are regulated by Ca^2+^ signaling. It seems likely, then, that transduction events mediated by plant apyrases would intersect with events mediated by Ca^2+^-regulated proteins in plants, such as calmodulin and annexin. In this regard, it is relevant to note that members of the annexin family of Ca^2+^ -binding proteins have been shown to play important roles in eATP signaling. For example, one of the earliest detectable eATP-induced signaling steps is an increase in the [Ca^2+^]_cyt_ and Annexin 1 facilitates this eATP-induced Ca^2+^ influx in Arabidopsis roots (Mohammad-Sidik et al. 2021). Annexins 2 and 4 also play roles in the eATP- and eADP-induced increases in [Ca^2+^]_cyt_ in Arabidopsis roots (Mohammad-Sidik 2019). Interestingly, the eATP-induced calcium signature in roots is diminished by phosphate starvation (Matthus et al. 2019).

In the Slocum et al. (2023) study previously discussed, transcriptomic analyses revealed that growth on Pi-deficient media induced an increase in transcript levels of Annexin 1 in both wild-type and *APY1*-OE seedlings. This upregulation of Annexin 1 expression in response to Pi starvation may occur to help restore normal eATP-mediated calcium signaling in roots. The Slocum et al. (2023) study also found that when seedlings were grown on Pi-sufficient media, NTP supplementation resulted in the downregulation of Annexins 3 and 4 in *APY1*-OE seedlings but not in wild-type seedlings.

Annexins also participate in auxin signaling mediated by apyrases and eATP in Arabidopsis. Annexin 3 helps regulate eATP-induced changes in seedling growth and polar localization of auxin transporters (Xu et al. 2023). Similarly, Annexins 1 and 2 play key roles in regulating PIN3 localization and auxin transport during seedling phototropic response (Wang et al. 2022). Further exploring the connections between annexins and apyrases in regulating eATP signaling would be a fertile area of future research.

Apyrase interactions with other proteins would also have important implications for their specific cellular functions. There is a critical need for more research identifying the protein partners that bind to apyrase and that may be required for its functions in different subcellular domains. Prior research has implicated some potential interactors, including ROP1-GTPase, which could partner with AtAPY1 in regulating pollen germination and elongation (Li et al. 2020), and a copper amine oxidase, which could interact with apyrases known to affect extracellular ROS levels during fungal attack (Toyoda et al. 2012). However, these initial studies should be confirmed and extended by independent methods such as yeast-two hybrid and co-IP assays.

## Post-transcriptional regulation of apyrase activity by inhibitors

Previous sections of this review focused on transgenic approaches (constitutive expression and knockouts) that have been used to gain insights on the roles of apyrases in regulating plant growth and development. Of course, apyrase expression can also be controlled post-transcriptionally, and a valuable approach to achieve this has been to use chemical inhibitors of apyrase. Windsor et al. (2002, 2003) reported an assay to find and test the effectiveness of such inhibitors. Using a colorimetric assay method, they assayed a library of small chemical compounds and identified several that could strongly inhibit potato APY. These compounds were relatively specific because they had less suppressive effect on the activity of other ATPases such as alkaline and acid phosphatases and luciferases (Windsor et al. 2002). The effectiveness of 2 of these inhibitors in enhancing the effectiveness of herbicides was demonstrated by increasing the [eATP], which suppressed the ability of ABC transporters to export the herbicides (Windsor et al. 2003), as previously described (Thomas et al. 2000).

These inhibitors were effective in blocking the activity of ecto-apyrases in diverse plants. As summarized in a prior review (Clark et al. 2021), treating Arabidopsis tissues or cotton ovules with these inhibitors mimicked the effects of genetically suppressing apyrase expression by increasing the [eATP] of growing cells and decreasing their growth rate (Wu et al. 2007). Treatment of cotton ovules with high [eATP] inhibits cotton fiber growth and increases ethylene production indicating that ethylene could also contribute to growth inhibition (Clark et al. 2010a). In Arabidopsis, both high [eATP] and genetic suppression of apyrase expression block auxin transport (Tang et al. 2003; Liu et al. 2012), inhibit the growth of pollen tubes (Wu et al. 2007), and suppress root growth and root skewing (Yang et al. 2015).

Apyrases are highly conserved throughout the evolution of eukaryotes, with at least 4 primary sequence domains that are highly similar in widely divergent organisms, including, for example, eudicots, ferns, and fungi (Clark et al. 2014). So, it was not surprising when Kumar Tripathy et al. (2017) found that the same inhibitors that suppressed growth in Arabidopsis also did so in species from 5 genera of pathogenic fungi, including *Botrytis, Colletotrichum, Sclerotinia, Fusarium*, and *Verticillium* (Kumar Tripathy et al. 2017). This result underscored the value of apyrase inhibitors in enhancing the potency of fungicides, just as they enhanced the potency of herbicides (Windsor et al. 2003). During infections, pathogens and pests improve the effectiveness of their attack on plants by secreting enzymes that lower the concentration of the eATP that plants use to induce their defense responses. For example, the pathogenic bacterium *Pseudomonas syringae* secretes NTPases as it is invading plant tissues. Pathogenic bacteria harboring a mutation that suppresses their ability to secrete these enzymes are unable to lower eATP levels and less able to infect plants (Tanaka and Heil 2021).

Herbivores such as the larvae of the corn earworm (*Helicoverpa zea*) and whitefly (*Bemisia tabaci*) secrete saliva containing ATP-hydrolyzing enzymes. At wound sites, these enzymes block the expression of defense associate genes. This is an evolutionarily developed function that pathogens and pests use to suppress the plant defense responses that are dependent on the eATP signal. Similarly, to promote their colonization of roots, symbiotic rhizobia and an endophytic fungus activate plant ectonucleotides and secrete their own nucleotidases that limit [eATP]. Parasitic worms and bloodsucking insects also follow similar mechanisms to regulate the eATP/ADP concentrations (Tanaka and Heil 2021).

More recently, Paes-Vieira et al. (2021) reported the major role of ecto-apyrases (e-NTPDases) in host-parasite interactions. *Leishmania amazonensis* infects the host cell by changing the immune response of macrophages, and it uses e-NTPDases to suppress the immune defense system of hosts by bringing down the level of ATP and ADP. This enzyme activity also produced AMP that was subsequently converted into adenosine, which then reduced the inflammatory response. During the parasite development, the expression of 2 genes encoding NTPDases, *ntpd1* and *ntpd2,* is differentially regulated. Promastigotes of *L. amazonensis* that overexpress either the *ntpd2* gene alone, or both *ntpd1* and *ntpd2* genes simultaneously, were more infective to macrophages than controls. Yet, mice that were transfected with parasites overexpressing *ntpd1* and *ntpd2* had fewer lesions than control mice. As explained by the authors, this contradictory effect of *ntpd1* and *ntpd2*, may be due to high levels of adenosine, and the activity of at least 2 different ecto-enzymes that hydrolyze nucleotides, e-NTPDase and ecto-5′-nucleotidase. The combined activity of these 2 enzymes would interfere with the balance of the immune response to promote the pathogen clearance and maintain the host protection. Overall, across multiple kingdoms, eATP evolved beyond just functioning as chemical energy to act as a danger signal that regulates many different cellular processes (Paes-Vieira et al. 2021).

The several apyrase inhibitors that have been used in plant publications so far (Clark et al. 2021) have amphipathic structures. This would allow them to cross cell membranes and thus inhibit not only ecto-apyrases, but also intracellular apyrases, including those in nuclei. It would be instructive to learn whether the effects of apyrase inhibitors on gene expression, like the effects of high [eATP] on auxin transport and organ growth, are similar to those observed in apyrase null mutants.

## Insights derived from recent data

The recent data discussed in this review provide insights on mechanisms by which apyrase could regulate growth. Proposals that apyrase impacts growth primarily by limiting [eATP] followed from observations that plants release ATP into their ECM as they grow, and that if the concentration of eATP was permitted to rise to levels above ∼ 500 *µ*m, this would likely inhibit the transport of auxin and negatively impact growth (Tang et al. 2003; Clark et al. 2021). A substantial rise in eATP can also inhibit growth by turning on jasmonic acid or ethylene signaling pathways (Lang et al. 2020; Shi et al. 2022).

The enhanced expression of apyrase in rapidly expanding tissues such as pollen tubes, etiolated hypocotyls, and the elongation zone of roots (Wu et al. 2007), plus the dramatic impact of apyrase suppression on growth inhibition (Wolf et al. 2007; Clark et al. 2010b; Liu et al. 2012) is consistent with the hypothesis that a major mechanism by which apyrase expression promotes growth is by helping to maintain [eATP] levels below those that decrease auxin transport or turn on jasmonic acid or ethylene production and thus inhibit growth. Those apyrases active in the ECM would be the ones most likely to directly affect [eATP], but, as noted above, Golgi-localized apyrase could also lower [eATP] by decreasing level in the secretory vesicles that deliver ATP to the ECM.

Of course, the extent of eATP induction of P2K1/P2K2 signaling pathways would be impacted by apyrase-mediated hydrolysis of eATP. However, the extent of this impact has not yet been adequately explored. Given the *K*_m_ of apyrase for ATP (∼30 *µ*m; Steinebrunner et al. 2000), and the very low threshold of [eATP] needed to activate the P2K1 and P2K2 receptors (*K*_d_ ∼ 45 nm; Choi et al. 2014; Pham et al. 2020), it is unlikely that ecto-apyrase hydrolysis of eATP would be able to limit very much the level of eATP available to activate these receptors. In fact, we would speculate that in transgenic plants overexpressing or ectopically expressing apyrase, the increase in growth observed might partially be attributed to the enzyme lowering the resting [eATP] to one that optimizes growth. This lower level would have to be below that needed to inhibit auxin transport or turn on hormones that inhibit growth, yet still high enough to activate the growth-promoting response of purinergic receptors.

By highlighting the gene expression changes induced by PS expression in Arabidopsis and soybeans, the reports of Veerappa et al. (2019), Sabharwal et al. (2022), and Weeraratne et al. (2022) bring more attention to the nuclear role of PS in growth control than did prior publications. Mechanistically, to the extent PS (and other plant APYs) can directly bind to DNA and induce changes in genes known to mediate auxin effects on growth (e.g. SAUR genes), or enhance the expression of genes that promote the uptake of nutrients, like phosphate, this nuclear function of apyrase would seem to link it more directly to growth control than would its ECM function of limiting [eATP]. The Golgi function of apyrases in protein glycosylation would also be expected to impact growth and development. However, hypotheses on how apyrases affect growth do not have to favor only 1 site of function. Given that high [eATP] inhibits auxin transport, the need for ecto-apyrases and/or Golgi apyrases to limit the build-up of eATP around expanding cells that release ATP as they grow could be as important as any growth-promoting gene expression changes induced by nuclear apyrases.

### Conclusions and unanswered questions

**Figure 1.**
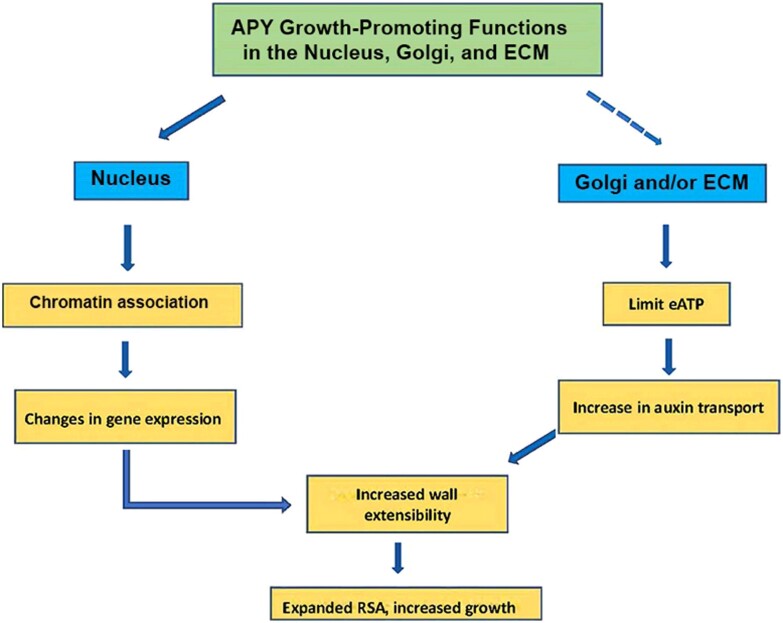
Summary of potential growth-related functions of apyrase (APY) in the nucleus, Golgi and the ECM. In Golgi, APY could also help to regulate glycosylation, and this, too, could impact growth. Current data indicate that in Arabidopsis some fraction of AtAPY1/2 is distributed in 1 or more of these subcellular compartments, but what cellular signals regulate this distribution is as yet unknown. Additionally, although a substantial portion of nuclear APY co-purifies with chromatin proteins, it is not yet known whether this is due to its association with other chromatin proteins or with DNA. RSA, root system architecture.

This review serves as an update to prior reviews, with a focus on those publications related to the regulation of growth by eATP and apyrases that have been published in the last 3 years. The recent work has further confirmed prior evidence, and expanded it by providing even more data consistent with the hypothesis that, in addition to ecto-apyrase and Golgi activities, the nuclear functions of apyrases could also play a major role in regulating the growth and development of plants (Fig. 1).

This review also highlighted unanswered questions that will require more research to answer (see Outstanding Questions and legend to Fig. 1). Experimental approaches in progress that could help answer these questions were discussed, but other, untried methodologies could also yield valuable insights. For example, single-cell proteomics and transcriptomics of seedling tissues (e.g. Clark et al. 2022) could demark more precisely those specific root or cotyledon cells that are expressing higher levels of apyrase and determine how closely these levels correspond with the growth rate of these cells. High resolution fluorescence microscopy methods, such as MINSTED nanoscopy (Weber et al. 2023) would be able to determine subnuclear APY localizations (nuclear membrane, nucleoplasm, chromatin?). The application of both classical and recently developed methods will undoubtedly provide unexpected and exciting answers to clarify the mechanisms by which apyrases regulate growth in plants.

ADVANCES BOX

- Early red light (R)-induced increases in cotyledon and root growth of etiolated seedlings occur with the same kinetics as R-induced enhanced accumulation of AtAPY1/2, and do not occur in *AtAPY1/2* mutants.
- In unirradiated seedlings, *AtAPY2* over-expression induces hook opening and increases expression of a *SAUR50* gene critical for R-induced cotyledon growth.
- Ectopic expression of a pea apyrase (PS) in greenhouse-grown Arabidopsis and soybean expands root system architecture (RSA), increases drought tolerance, and turns on genes that promote root growth and drought tolerance.
- Ectopic expression of PS in field-grown soybean significantly increases seed yield in multiple sites.
- Constitutive expression of *AtAPY1* enhances Pi uptake and RSA expansion in seedlings, turns on the expression of genes that promote auxin transport, and increases Pi scavenging from eNTPs.

OUTSTANDING QUESTIONS BOX

- What environmental or cellular signals determine the relative distribution of AtAPY1/2, PS, and other growth-regulating apyrases in Golgi, ECM, or nucleus?
- What is the subnuclear site where growth-regulating apyrases function?
- Are the effects of AtAPY1/2, PS, and other growth-regulating apyrases on gene expression due mainly to their function in Golgi, ECM, or nucleus?
- What post-translational modifications (e.g. glycosylation, phosphorylation) are made to plant apyrases, and what are the effects of these modifications on in vivo enzyme activity?
- What proteins associate with growth-regulating apyrases, and how do they contribute to apyrase function?
- How critical is Ca^2+^-activated calmodulin and annexins to the function of apyrases for growth regulation?

## Dive Curated Terms

The following phenotypic, genotypic, and functional terms are of significance to the work described in this paper:

NIH Gramene: AT1G06670
NIH Araport: AT1G06670

PIN3 Gramene: AT1G70940
PIN3 Araport: AT1G70940

AtAPY1 Gramene: AT3G04080
AtAPY1 Araport: AT3G04080

AtAPY2 Gramene: AT5G18280
AtAPY2 Araport: AT5G18280

TGG2 Gramene: AT5G25980
TGG2 Araport: AT5G25980

ADP CHEBI: CHEBI:16761

CAMTA3 Gramene: AT2G22300
CAMTA3 Araport: AT2G22300
